# Localized, highly efficient secretion of signaling proteins by migrasomes

**DOI:** 10.1038/s41422-024-00992-7

**Published:** 2024-06-25

**Authors:** Haifeng Jiao, Xiaopeng Li, Ying Li, Yuting Guo, Xiaoyu Hu, Takami Sho, Yiqun Luo, Jinyu Wang, Huizhen Cao, Wanqing Du, Dong Li, Li Yu

**Affiliations:** 1 State Key Laboratory of Membrane Biology, Tsinghua University-Peking University Joint Centre for Life Sciences, Beijing Frontier Research Center for Biological Structure, School of Life Sciences, Tsinghua University, Beijing, China; 2 National Laboratory of Biomacromolecules, CAS Center for Excellence in Biomacromolecules, Institute of Biophysics, Chinese Academy of Sciences, Beijing, China; 3 SLSTU-Nikon Biological Imaging Center, Center of Biomedical Analysis, Tsinghua University, Beijing, China

**Keywords:** Organelles, Cell migration

## Abstract

Migrasomes, enriched with signaling molecules such as chemokines, cytokines and angiogenic factors, play a pivotal role in the spatially defined delivery of these molecules, influencing critical physiological processes including organ morphogenesis and angiogenesis. The mechanism governing the accumulation of signaling molecules in migrasomes has been elusive. In this study, we show that secretory proteins, including signaling proteins, are transported into migrasomes by secretory carriers via both the constitutive and regulated secretion pathways. During cell migration, a substantial portion of these carriers is redirected to the rear of the cell and actively transported into migrasomes, driven by the actin-dependent motor protein Myosin-5a. Once at the migrasomes, these carriers fuse with the migrasome membrane through SNARE-mediated mechanisms. Inhibiting migrasome formation significantly reduces secretion, suggesting migrasomes as a principal secretion route in migrating cells. Our findings reveal a specialized, highly localized secretion paradigm in migrating cells, conceptually paralleling the targeted neurotransmitter release observed in neuronal systems.

## Introduction

Migrasomes, recently identified organelles in migrating cells, form on retraction fibers, which are elongated membrane tethers present at the cell’s trailing edge. These migrasomes, characterized by their large vesicular structure with diameters averaging around 2 µm, are notable for housing numerous intraluminal vesicles, the origins of which remain largely unknown.^1^ Migrasomes play an instrumental role in the targeted delivery of signaling molecules, including chemokines and cytokines, to precise locations, thereby exerting a substantial influence on physiological processes that require the integration of spatial and chemical information. A prominent example is observed during the gastrulation phase of zebrafish embryonic development, where migrasomes formed by mesendodermal cells are enriched with signaling ligands such as chemokines and growth factors, contributing significantly to the process of organ morphogenesis.^2^ Similarly, recent investigations have demonstrated that during embryonic angiogenesis, migrating monocytes in chicken embryos release migrasomes enriched with vascular endothelial growth factor A (VEGFA) along their migratory tracks, thereby orchestrating capillary growth and playing a critical role in angiogenesis.^3^ The precise mechanisms underlying the selective transport and subsequent release of these signaling ligands from migrasomes, however, remain to be fully elucidated.

Cellular secretion, a fundamental biological process, commences with the guiding of secretory proteins to the endoplasmic reticulum (ER) by signal peptides. Following translocation into the ER lumen, where signal peptides are cleaved, these proteins are ferried to the Golgi complex via COPII-coated vesicles.^4–6^ Within the Golgi, they undergo further post-translational modifications before being routed for release through either the constitutive or regulated secretory pathways. The regulated pathway, in particular, requires granule or organelle intermediates for secretion, and is activated by specific cellular signals.^7^ In this context, recycling endosomes, especially their tubular protrusions rich in VAMP3,^8,9^ are crucial for certain Rab GTPases, such as Rab11, to regulate the targeted delivery of secretory cargoes like TNF to the plasma membrane.^10,11^ Rab8, in contrast, is typically associated with the constitutive secretory pathway.^12^

Motor proteins, encompassing kinesins, dyneins and myosins, are pivotal in the intracellular transport of secretory vesicles. These proteins traverse the cytoskeletal network, with kinesins and dyneins facilitating long-range transport along microtubules, and myosins, particularly Myosin-5a, orchestrating the short-range movement of vesicles along actin filaments.^13–16^ This is crucial for the final steps of secretion, including vesicle docking and fusion with the plasma membrane.^17,18^ The final fusion of secretory carriers with the plasma membrane is mediated by Q-SNAREs such as SNAP23, and by R-SNAREs such as VAMP2 and VAMP3, which have emerged as useful markers of secretory carriers.^19,20^ Various studies have shown that the level of SNARE proteins is the rate-limiting step of cytokine secretion. The efficiency and volume of secretion is modulated by the level of SNARE proteins on the membrane; thus, the membrane density of SNARE proteins determines the propensity for secretion.^21,22^

Highly localized secretion is well-documented in neurons. In these cells, synaptic vesicles containing neurotransmitters are transported to axon terminals via axonal transport, driven by motor proteins. Upon arrival, the vesicles are primed for release, awaiting an action potential to trigger neurotransmitter release at the axon terminal — a critical step for synaptic transmission.^23–25^ The extent to which other cell types exhibit similar specialized secretion mechanisms remains an open question. The occurrence of such processes in specialized cellular structures other than axon terminals is less understood.

In this study, we demonstrate that secretory proteins, including signaling proteins, are transported into migrasomes via secretory carriers from both constitutive and regulated secretion pathways. We found that during cell migration, a significant portion of these carriers is redirected to the rear of the cell and actively transported into migrasomes. This process is driven by the actin-dependent motor protein Myosin-5a. Additionally, we show that these carriers fuse with the migrasome membrane through SNARE-mediated mechanisms. Significantly, our research also reveals that inhibiting migrasome formation drastically reduces secretion, suggesting that migrasomes are a principal secretion route in migrating cells. On the basis of these data, we propose a previously unknown secretion paradigm in migrating cells, analogous to synaptic vesicle release.

## Results

### Characterization of the intraluminal vesicles of migrasomes

**Fig. 1:**
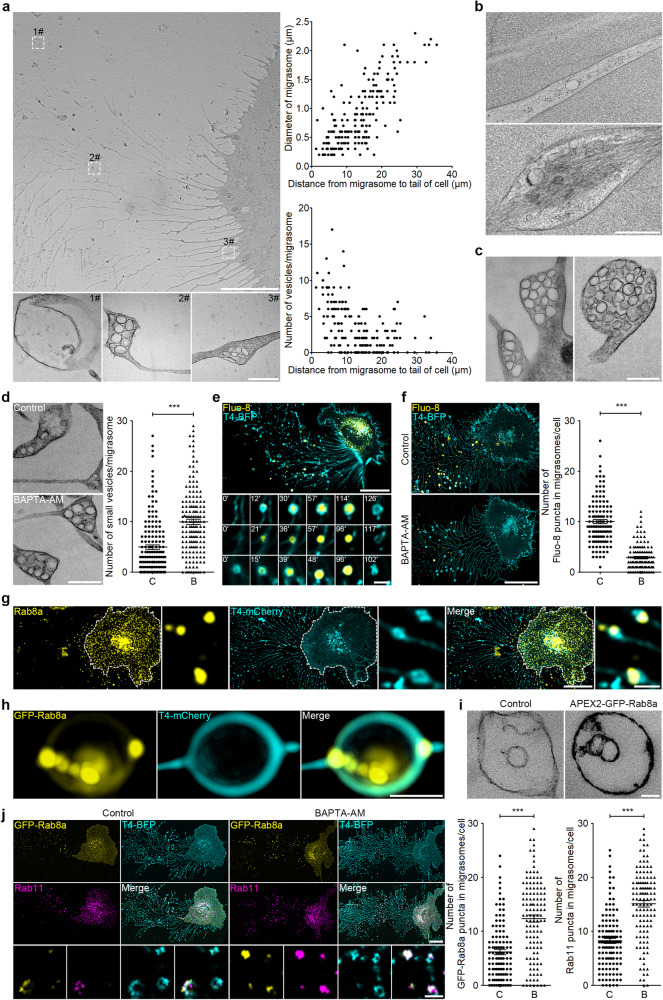
Characterization of the intraluminal vesicles of migrasomes. **a** TEM images of an L929 cell. Scale bar, 10 μm. Lower panels, enlarged regions of interest (ROI). Scale bar, 500 nm. Right panels, quantification of the relationship between the distance from the migrasome to the cell body and the migrasome diameter (top) or the number of intraluminal vesicles per migrasome (bottom). *n* = 30 cells from three independent experiments. **b** TEM images of high-pressure freezing samples of retraction fiber and migrasome from L929 cells. Upper panel, retraction fiber. Lower panel, migrasome. Scale bar, 500 nm. **c** TEM images of migrasomes from L929 cells. Left panel, the entrances of retraction fibers. Right panel, a detached migrasome. Scale bar, 500 nm. **d** TEM images of migrasomes from L929 cells treated with 10 μM BAPTA-AM for 10 h. Scale bar, 500 nm. Right panel, statistical analysis of the number of small vesicles per migrasome. Data are means ± SEM. C control, B BAPTA-AM. *n* > 100 migrasomes from three independent experiments. Two-tailed unpaired *t*-test was used for statistical analyses. ***P < 0.001. **e** L929 cells stably expressing Tspan4 (T4)-BFP were stained with Fluo-8 and then subjected to time-lapse imaging. Time interval, 180 s. Scale bar, 20 μm. The lower panels show enlarged migrasomes. Scale bar, 2 μm. **f** L929-T4-BFP cells, treated with 10 μM BAPTA-AM for 10 h, were stained with Fluo-8 and then visualized. Scale bar, 20 μm. Right panel, statistical analysis of the number of Fluo-8 puncta in migrasomes per cell. Data are means ± SEM. *n* > 100 cells from three independent experiments. Two-tailed unpaired *t*-test was used for statistical analyses. ***P < 0.001. **g** L929-T4-mCherry cells were immunostained with antibody against Rab8a and then visualized. White dashed lines outline the cell body. Scale bar, 20 µm. Right panels, enlarged ROI. Scale bar, 2 µm. **h** SIM images of a migrasome from L929 cells stably expressing GFP-Rab8a and T4-mCherry. Scale bar, 500 nm. **i** Representative TEM images of the DAB staining pattern in migrasomes from L929-APEX2-GFP-Rab8a cells. Scale bar, 100 nm. **j** GFP-Rab8a- and T4-BFP-expressing L929 cells, treated with 10 μM BAPTA-AM for 10 h, were stained with Rab11 antibody and then visualized. Scale bar, 20 μm. Lower panels, enlarged ROI. Scale bar, 2 μm. Right panels, statistical analysis of the number of GFP-Rab8a and Rab11 puncta in migrasomes per cell. Data are means ± SEM. *n* > 100 cells from three independent experiments. Two-tailed unpaired *t*-test was used for statistical analyses. ***P < 0.001.

Previously we reported the presence of intraluminal vesicles in migrasomes. To carry out in-depth investigation of the origin and identity of these vesicles, we first examined migrasome-forming cells by transmission electron microscopy (TEM). We found that the size of migrasomes and the number of intraluminal vesicles are closely correlated with the distance between migrasomes and the cell body: migrasomes further away from cells are larger and contain fewer intraluminal vesicles (Fig. 1a). We also found individual or small clusters of intraluminal vesicles in retraction fibers. In many cases, actin filaments are visibly associated with these vesicles (Fig. 1b). Moreover, we observed large clusters of vesicles on the base of retraction fibers and in detached migrasomes (Fig. 1c). The positioning and the distribution of these vesicles suggest that the intraluminal vesicles of migrasomes may be transported to the base of the retraction fiber, and then transported to migrasomes via retraction fibers.

The observation that distal migrasomes contain fewer intraluminal vesicles suggests that these vesicles may fuse with the migrasome membrane. Calcium is known to be essential for SNARE-mediated vesicle fusion.^26^ In our experiments, treating cells with BAPTA-AM, a cell-permeant calcium chelator, led to a significant increase in the number of intraluminal vesicles in migrasomes (Fig. 1d). To further investigate the potential role of calcium signals in regulating secretion, we performed calcium imaging using the fluorescent indicator Fluo-8. We observed a continuous calcium signal within the migrasome during its formation. In the early stages of migrasome formation, the calcium signal was faint and appeared to be localized to small dots along the migrasome’s edge. As the migrasome matured, the intensity of the calcium signal got amplified, eventually pervading the entire migrasome lumen (Fig. 1e; Supplementary information, Video S1). When cells were treated with BAPTA-AM, the calcium signal within the migrasome disappeared (Fig. 1f). Collectively, these findings support our hypothesis that intraluminal vesicles may fuse with the migrasome membrane in a calcium-dependent manner.

### Rabs mark migrasome intraluminal vesicles

Our previous mass spectrometry analysis identified that Rab8 is enriched in migrasomes. Rab8 has been reported to regulate Golgi-to-plasma membrane trafficking in constitute exocytosis.^27^ We found that endogenous Rab8a is indeed present inside migrasomes and along the retraction fibers (Fig. 1g). Structured illumination microscopy (SIM) images showed that GFP-Rab8a signals are present as intraluminal puncta inside migrasomes (Fig. 1h). To check whether these puncta are intraluminal vesicles, we carried out APEX2-based intracellular-specific protein imaging by electron microscopy (EM). APEX2 catalyzes the local deposition of diaminobenzidine (DAB), which enhances the contrast in EM images by binding electron-dense osmium. We found that APEX2-GFP-Rab8a is indeed localized on the intraluminal vesicles of migrasome (Fig. 1i). Thus, the intraluminal vesicles are positive for Rab8a. Moreover, we found that BAPTA-AM significantly increased the number of GFP-Rab8a-labeled vesicles in migrasomes, which is consistent with our TEM analysis (Fig. 1j). In addition to Rab8a, we also found that Rab11, a marker for the recycling endosome and known for its pivotal role in TNF-α secretion, is enriched in migrasomes. Moreover, Rab5- and Rab10-labeled vesicles are also present in migrasomes. Similar to Rab8a, BAPTA-AM treatment significantly increased the number of Rab11-, Rab5- and Rab10-labeled vesicles in migrasomes (Fig. 1j; Supplementary information, Fig. S1a, b). Together, these data suggest that secretory carriers from various secretion routes can be transported into the migrasome.

### SNAREs mediate the fusion of intraluminal vesicles with migrasome membrane

**Fig. 2:**
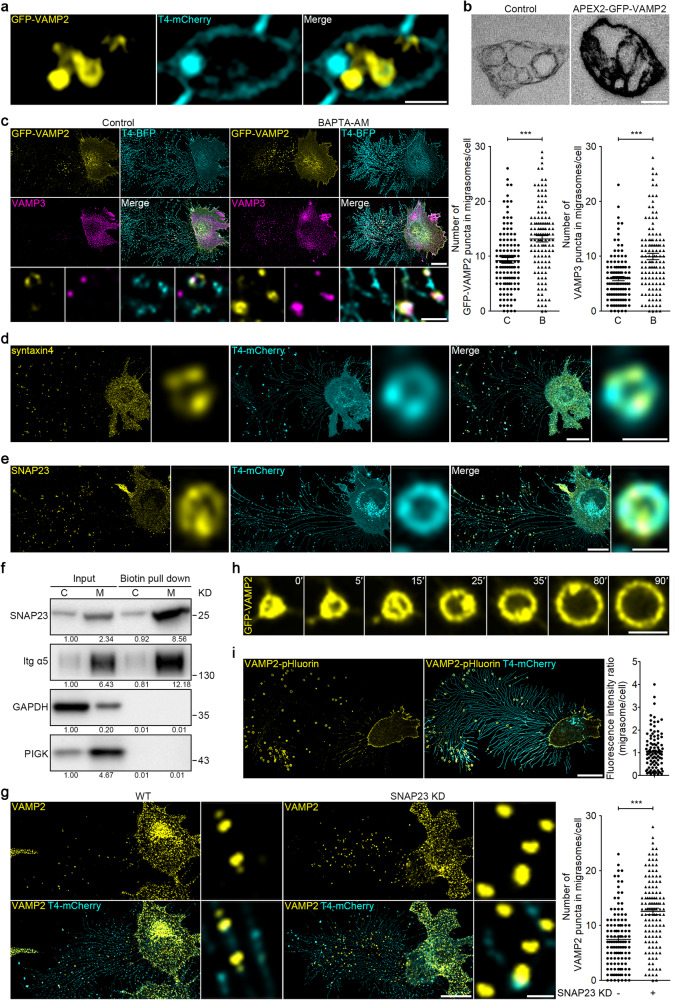
SNAREs mediate the fusion of intraluminal vesicles with the migrasome membrane. **a** SIM images of L929 cells stably expressing GFP-VAMP2 and T4-mCherry. Scale bar, 200 nm. **b** TEM images of L929 cells stably expressing APEX2-GFP-VAMP2 and reacted with DAB. Scale bar, 200 nm. **c** L929 cells stably expressing GFP-VAMP2 and T4-BFP, treated with or without 10 μM BAPTA-AM for 10 h, were stained with VAMP3 antibody and then visualized. Scale bar, 20 μm. Lower panels, enlarged ROI. Scale bar, 2 µm. Right panels, statistical analysis of the number of GFP-VAMP2 and VAMP3 puncta in migrasomes per cell. Data are means ± SEM. *n* > 100 cells from three independent experiments. Two-tailed unpaired *t*-test was used for statistical analyses. ***P < 0.001. **d**, **e** Immunostaining of endogenous syntaxin4 (**d**) or SNAP23 (**e**) in L929-T4-mCherry cells. Scale bar, 20 µm. The right panels show enlarged migrasomes. Scale bar, 1 μm. **f** L929 cells were cultured in FN-precoated dishes for 10 h, and were then treated with Sulfo-NHS-SS-Biotin to biotinylated membrane proteins. Biotin-labeled membrane proteins were subsequently isolated from cell bodies or migrasomes using NeutrAvidin Agarose, respectively. Equal amounts of total protein from cell bodies (C) or migrasomes (M) were then subjected to western blot analysis. Integrin α5 (Itg α5) and PIGK were used as migrasome markers in L929 cells. Representative densitometry analysis of western blot gray values is shown. Three independent experiments were conducted. **g** Immunostaining of endogenous VAMP2 in wild-type (WT) or SNAP23 KD L929-T4-mCherry cells. Scale bar, 20 µm. Right panels, enlarged ROI. Scale bar, 2 µm. Statistical analysis of the number of VAMP2 puncta in migrasomes per cell is shown as the means ± SEM. *n* > 100 cells from three independent experiments were analyzed using the two-tailed unpaired *t*-test. ***P < 0.001. **h** L929 cells stably expressing GFP-VAMP2 were subjected to time-lapse imaging. Time-lapse images were acquired at intervals of 30 s. Scale bar, 2 µm. **i** Confocal images of L929 cells stably expressing VAMP2-pHluorin and T4-mCherry. Scale bar, 20 µm. The right panel shows statistical analysis of the fluorescence intensity ratio. Each point represents the migrasome (all the migrasomes from an individual cell)/cell body fluorescence intensity ratio. *n* > 100 cells from three independent experiments.

Next, we investigated the SNAREs required for the fusion of intraluminal vesicles with migrasome membranes. We found that VAMP2, a v-SNARE involved in constitutive exocytosis, is localized inside migrasomes as small puncta (Fig. 2a). Not surprisingly, APEX2-based TEM revealed that VAMP2 is localized on the membrane of intraluminal vesicles (Fig. 2b). Moreover, we found that BAPTA-AM treatment significantly increased the number of VAMP2- and VAMP3-labeled vesicles in migrasomes (Fig. 2c). Knocking down VAMP2 led to a significant increase in the number of Rab8a vesicles in migrasomes, which indicates that VAMP2 is required for the fusion of Rab8a vesicles with migrasome membrane (Supplementary information, Fig. S2a). In non-neuron cells, VAMP2 mediates exocytosis mainly by forming a SNARE complex with the t-SNAREs, including syntaxin4 (Qa) and SNAP23 (Qbc).^28^ We found that both syntaxin4 and SNAP23 are localized on migrasomes (Fig. 2d, e; Supplementary information, Fig. S2b). To confirm this observation biochemically, we isolated the total membrane proteins from plasma membranes and from migrasomes. We found that SNAP23 is markedly enriched in migrasomes (Fig. 2f). Finally, syntaxin4 or SNAP23 knockdown significantly increased the number of VAMP2 vesicles in migrasomes, which suggests that VAMP2 vesicles undergo fusion with migrasome membranes in a syntaxin4- and SNAP23-dependent manner (Fig. 2g; Supplementary information, Fig. S2c). Taken together, these data suggest that intraluminal vesicles may fuse with migrasomes through SNAREs.

To directly visualize the fusion of VAMP2 vesicles with the migrasome membrane, we carried out time-lapse imaging. The VAMP2 signal starts as a cluster of small puncta; as the migrasome grows, the VAMP2 signal gradually moves to the migrasome membrane, which suggests that fusion has occurred (Fig. 2h; Supplementary information, Video S2). To directly detect the fusion between VAMP2 vesicles and migrasome membranes, we generated a VAMP2-pHluorin-expressing cell line. pHluorin is a pH-sensitive green fluorescent protein which has been widely used to visualize vesicle secretion. In cells labeled with VAMP2-pHluorin, there are no vesicular signals inside cells or inside migrasomes, as these vesicles are acidic; instead, all the signals are on the plasma membrane or on the migrasome membranes. Importantly, the VAMP2-pHluorin signal is more intense on migrasome membranes than on the plasma membrane of the cell body in an observation plane (Fig. 2i). This suggests that in these cells, the migrasome is the preferred secretion site compared to the plasma membrane. Taken together, these data suggest that VAMP2 vesicles fuse with migrasomes, and in some cells, migrasomes appear to be the preferred sites of fusion for VAMP2 vesicles.

### Myosin-5a is actively transported into the migrasome

**Fig. 3:**
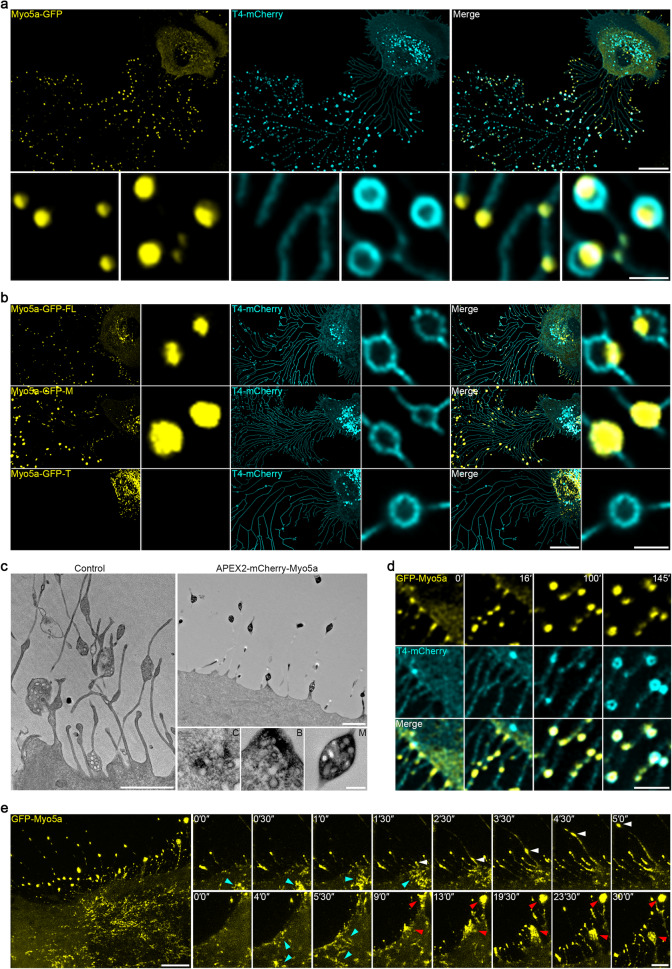
Myosin-5a is actively transported into the migrasome. **a** Confocal images of L929 cells stably expressing Myosin-5a (Myo5a-GFP) and T4-mCherry. Scale bar, 20 µm. Lower panels, enlarged ROI. Scale bar, 2 µm. **b** Confocal images of L929-T4-mCherry cells stably expressing the indicated forms of Myo5a: full-length (FL), motor domain (M) and tail domain (T). Scale bar, 20 µm. Right panels, enlarged ROI. Scale bar, 2 µm. **c** APEX2-based TEM images of L929 cells stably expressing APEX2-mCherry-Myo5a. Scale bar, 2 µm. The lower panels show higher-magnification images of vesicles from the cell body (C), the base of a retraction fiber (B) and the migrasome (M). Scale bar, 200 nm. **d** Time-lapse images of L929 cells stably expressing GFP-Myo5a and T4-mCherry. Time interval, 90 s. Scale bar, 5 µm. **e** GI-SIM images of L929-GFP-Myo5a cells. Time-lapse images were acquired at intervals of 30 s. Scale bar, 5 µm. Right panels, enlarged ROI. Blue arrowheads indicate Myo5a transporting to the edge of the cell. White arrowheads indicate Myo5a moving into migrasomes. Red arrowheads indicate Myo5a accumulating at the edge of cell and left on retraction fibers. Scale bar, 2 µm.

Next, we sought to identify the motor proteins which may transport intraluminal vesicles to migrasomes. Since we observed bundled actin inside retraction fibers, we focused our search on the actin-based motor proteins, namely myosins. Among them, we were particularly interested in Myosin-5a, which is known for mediating long-distance transportation of vesicles. To check the localization of Myosin-5a, we generated a cell line in which Myosin-5a-GFP is stably expressed. We found that Myosin-5a-GFP forms bright puncta along retraction fibers and inside migrasomes, and the signals from these puncta are much brighter than the signals inside cells (Fig. 3a). Moreover, GFP fused to the Myosin-5a motor domain is highly enriched in migrasomes, while GFP fused to the Myosin-5a tail domain is absent from migrasomes (Fig. 3b). This suggests that the motor domain is required for localization of Myosin-5a in migrasomes. APEX2-based TEM imaging revealed that APEX2-mCherry-Myosin-5a is indeed decorated around intraluminal vesicles and the clustered vesicles at the base of retraction fibers (Fig. 3c). This suggests that intraluminal vesicles may be transported to the base of retraction fibers and into migrasomes by Myosin-5a. To visualize the movement of intraluminal vesicles, we carried out time-lapse imaging. This showed that the GFP-Myosin-5a signals on retraction fibers increased when the cell migrated away, eventually becoming bright puncta. In many cases, migrasomes grew around the GFP-Myosin-5a puncta, so that the GFP-Myosin-5a puncta were eventually enclosed in migrasomes (Fig. 3d; Supplementary information, Video S3). These data indicated that GFP-Myosin-5a is transported to the site of migrasome formation. Moreover, the gradual increase in the GFP-Myosin-5a signal at migrasome formation sites suggests that GFP-Myosin-5a may be gradually transported to these sites. To better characterize the movement of Myosin-5a, we carried out ultra-fast super-resolution imaging using grazing incidence-structured illumination microscopy (GI-SIM), which can reach 100 nm resolution with a speed of 200 frames/s. GI-SIM imaging showed that small Myosin-5a puncta are transported to the edge of the cell, where they accumulate as bright clusters. From these clusters on the base of retraction fibers, a stream of small vesicles rapidly moves into migrasomes. In some cases, clusters of vesicles are left on retraction fibers when cells migrate away (Fig. 3e; Supplementary information, Video S4). Taken together, these data suggest that intraluminal vesicles may be transported into migrasomes by Myosin-5a.

### Myosin-5a mediates transport of migrasome intraluminal vesicles

**Fig. 4:**
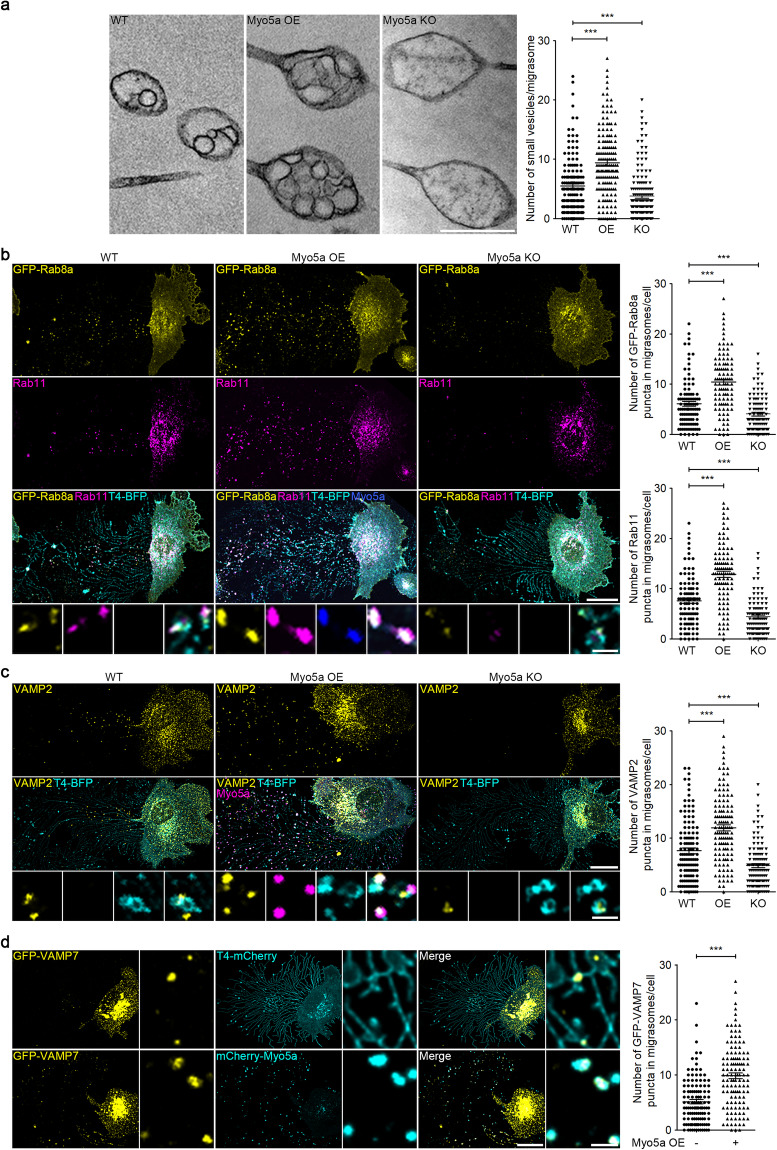
Myosin-5a mediates transport of migrasome intraluminal vesicles. **a** TEM images of WT, Myo5a OE and Myosin-5a knockout (Myo5a KO) L929 cells. Scale bar, 500 nm. The right panel shows statistical analysis of the number of small vesicles per migrasome. Data are means ± SEM for > 100 migrasomes from three independent experiments. Two-tailed unpaired *t*-test was used for statistical analyses. ***P < 0.001. **b** Immunostaining of endogenous Rab11 in WT, Myo5a OE and Myo5a KO L929-GFP-Rab8a cells. Scale bar, 20 μm. Lower panels, enlarged ROI. Scale bar, 2 μm. Right panels, statistical analysis of the number of GFP-Rab8a and Rab11 puncta in migrasomes per cell. Data are means ± SEM. *n* > 100 cells from three independent experiments. Two-tailed unpaired *t*-test was used for statistical analyses. ***P < 0.001. **c** Immunostaining of endogenous VAMP2 in WT, Myo5a OE and Myo5a KO L929 cells. Scale bar, 20 µm. Lower panels, enlarged ROI. Scale bar, 2 μm. Right panel, statistical analysis of the number of VAMP2 puncta in migrasomes per cell. Data are means ± SEM. *n* > 100 cells from three independent experiments. Two-tailed unpaired *t*-test was used for statistical analyses. ***P < 0.001. **d** Stable expression of T4-mCherry or mCherry-Myo5a was established in L929-GFP-VAMP7 cells. The cells were then subjected to confocal analysis. Scale bar, 20 µm. Right panels, enlarged ROI. Scale bar, 2 µm. Statistical analysis of the number of GFP-VAMP7 puncta in migrasomes per cell is shown as the means ± SEM. *n* > 100 cells from three independent experiments were analyzed using the two-tailed unpaired *t*-test (right panel). ***P < 0.001.

To directly test this hypothesis, we established cell lines in which Myosin-5a is stably overexpressed (Myo5a OE) or knocked out (Myo5a KO), and checked the number of intraluminal vesicles by TEM. We found that overexpression of Myosin-5a increased while knockout of Myosin-5a decreased the number of intraluminal vesicles (Fig. 4a). This suggests that intraluminal vesicles are indeed transported into migrasomes by Myosin-5a. Similarly, Myosin-5a overexpression significantly increased while Myosin-5a knockout markedly reduced the number of Rab8a, Rab11 and VAMP2 puncta in migrasomes, which indicates that Rab8a-, Rab11- and VAMP2-positive vesicles are transported into migrasomes by Myosin-5a (Fig. 4b, c). Moreover, we also found that Myosin-5a mediates transport of Rab5-, Rab10- and VAMP7-labeled vesicles into migrasomes, which suggests that migrasomes can release secretory vesicles from different traffic route in a Myosin-5a-dependent manner (Fig. 4d; Supplementary information, Fig. S3a, b).

### Cell migration causes the polarization of secretory carriers to the rear end of the cell

**Fig. 5:**
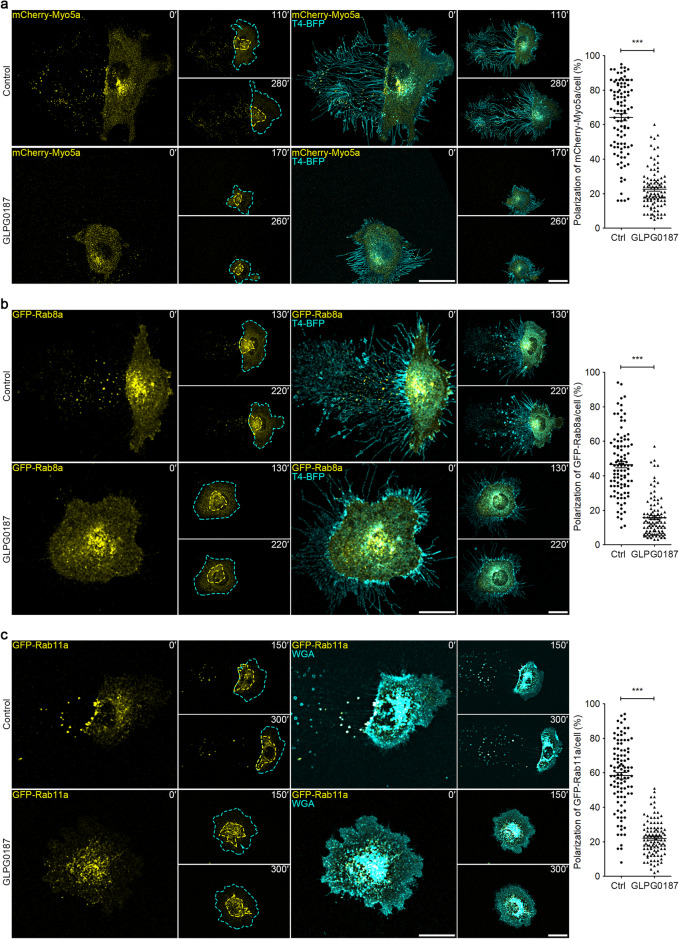
Cell migration causes the polarization of secretory carriers to the rear end of the cell. **a** L929 cells stably expressing mCherry-Myo5a and T4-BFP, treated with or without 10 μM GLPG0187, were subjected to time-lapse imaging. Time interval, 10 min. Cyan dashed lines outline the cell body, and yellow dashed lines outline mCherry-Myo5a puncta. Scale bar, 20 μm. Polarization of mCherry-Myo5a was quantified and shown as the means ± SEM for triplicate samples of > 50 cells. Two-tailed unpaired *t*-test was used for statistical analyses (right panel). ***P < 0.001. **b** L929 cells stably expressing GFP-Rab8a and T4-BFP, treated with or without 10 μM GLPG0187, were subjected to time-lapse imaging. Time interval, 10 min. Cyan dashed lines outline the cell body, and yellow dashed lines outline GFP-Rab8a vesicles, respectively. Scale bar, 20 μm. Polarization of GFP-Rab8a was quantified and shown as the means ± SEM for triplicate samples of > 50 cells. Two-tailed unpaired *t*-test was used for statistical analyses (right panel). ***P < 0.001. **c** L929 cells stably expressing GFP-Rab11a, treated with or without 10 μM GLPG0187, were stained with wheat germ agglutinin (WGA) and then subjected to time-lapse imaging. Time interval, 10 min. Cyan dashed lines outline the cell body, and yellow dashed lines outline GFP-Rab11a vesicles, respectively. Scale bar, 20 μm. Polarization of GFP-Rab11a was quantified and shown as the means ± SEM for triplicate samples of > 50 cells. Two-tailed unpaired *t*-test was used for statistical analyses (right panel). ***P < 0.001.

We hypothesized that if secretory carriers are transported into migrasomes, which are localized at the rear end of the cell, then the trafficking route of these carriers might be polarized toward the cell’s rear in migrating cells. To test this hypothesis, we engineered L929 cells to stably express mCherry-Myosin-5a, GFP-Rab8a and GFP-Rab11a. We observed that in migrating cells, the majority of the mCherry-Myosin-5a, GFP-Rab8a and GFP-Rab11a signals were localized at the cell’s rear, with only a minimal presence at the front (Fig. 5a–c; Supplementary information, Videos S5–S7). This polarization of vesicles was largely abolished when cell migration was inhibited by treatment with GLPG0187, a broad-spectrum integrin receptor antagonist (Fig. 5a–c; Supplementary information, Videos S5–S7). Collectively, these data suggest that cell migration can reroute the trafficking of secretory carriers to the rear of cells.

### Migrasomes are enriched with cytokines

**Fig. 6:**
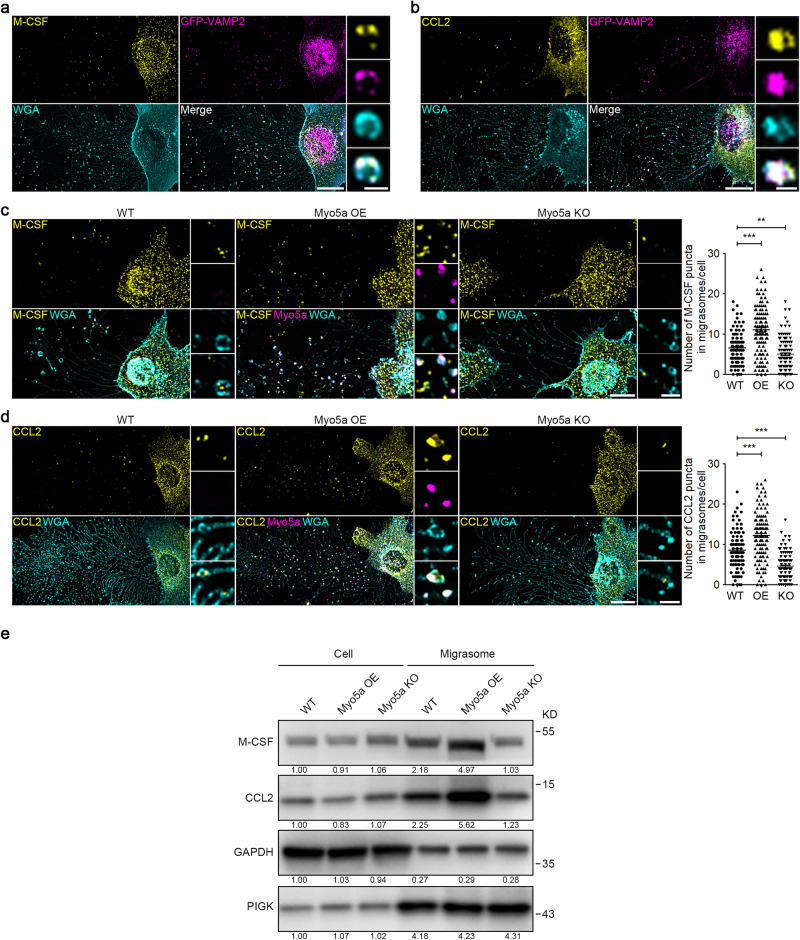
Migrasomes are enriched with cytokines. **a**, **b** L929-GFP-VAMP2 cells were stained with M-CSF (**a**) or CCL2 (**b**) antibody and then visualized. Scale bar, 20 μm. The right panels show enlarged migrasomes. Scale bar, 2 μm. **c**, **d** Immunostaining of endogenous M-CSF (**c**) or CCL2 (**d**) in WT, Myo5a OE and Myo5a KO L929 cells. Scale bar, 20 µm. Right panels, enlarged ROI. Scale bar, 2 µm. Statistical analysis of the number of M-CSF (**c**) and CCL2 (**d**) puncta in migrasomes per cell is shown as the means ± SEM. *n* > 100 cells from three independent experiments were analyzed using the two-tailed unpaired *t*-test (right panel). **P < 0.01, ***P < 0.001. **e** Migrasomes were purified from the indicated L929 cells. Cell lysates and migrasomes were normalized with total protein and subjected to western blot analysis using the indicated antibodies. PIGK was used as a migrasome marker in L929 cells. Representative densitometry analysis of western blot gray values is shown. Three independent experiments were conducted.

Secretory proteins are released from cells through the fusion of secretory vesicles with the plasma membrane. Thus, we next investigated whether secretory proteins, such as cytokines and chemokines, are transported into migrasomes by secretory vesicles. Secretome analysis revealed that L929 cells secrete cytokines, including macrophage colony-stimulating factor (M-CSF) and monocyte chemoattractant protein-1 (also known as chemokine (C-C motif) ligand 2 (CCL2)).^29^ By staining cells with antibodies against M-CSF or CCL2 in L929-GFP-VAMP2 cells, we observed that these cytokines are indeed present in migrasomes co-localizing with VAMP2 puncta, which revealed that both cytokines are transported into migrasomes by secretory vesicles (Fig. 6a, b). Furthermore, overexpression of Myosin-5a significantly increases, while knockout of Myosin-5a notably decreases, the M-CSF and CCL2 signals in migrasomes (Fig. 6c, d). This lends further support to the notion that both cytokines are transported into migrasomes via the Myosin-5a-mediated transportation. Next, we checked whether M-CSF and CCL2 are enriched in migrasomes. Western blot analysis of cell bodies and migrasomes revealed that both M-CSF and CCL2 are indeed enriched in migrasomes. Moreover, overexpressing Myosin-5a enhances while knocking out Myosin-5a reduces the amount of M-CSF and CCL2 in migrasomes (Fig. 6e). This provides further evidence that both cytokines are transported into migrasomes by Myosin-5a-mediated transportation of secretory vesicles.

To determine whether secretory carriers are transported into migrasomes in other cell types, we examined dental pulp stem cells (DPSCs), a primary cell line. We found that Rab8a, Rab11, VAMP2, VAMP3 and a secretory protein, VEGFA, are also enriched in migrasomes (Supplementary information, Fig. S4). This indicates that this pathway is not restricted to L929 cells.

### Migrasome formation dictates the amount of cytokines released into the medium

**Fig. 7:**
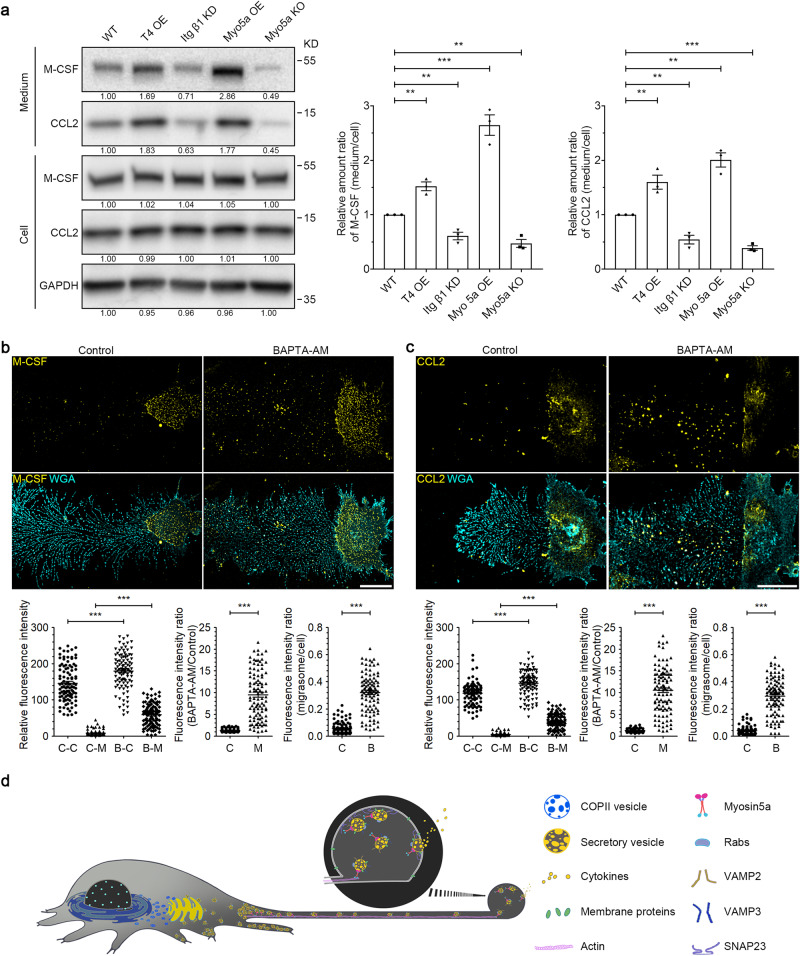
Migrasomes are the major sites of secretion in migrating cells. **a** Secretion analysis of M-CSF and CCL2 in the indicated L929 cells. The concentrated media were collected and normalized with the numbers of cells, and were then subjected to western blot analysis using the indicated antibodies. Representative densitometry analysis of western blot gray values is shown. The ratios of secreted cytokines vs those in the cell body were quantified and shown as the means ± SEM from three independent experiments. Two-tailed unpaired *t*-test was used for statistical analyses (lower panels). **P < 0.01, ***P < 0.001. **b**, **c** L929 cells, either untreated or treated with 10 μM BAPTA-AM for 10 h, were stained with M-CSF (**b**) or CCL2 (**c**) antibody. *Z*-stack images were acquired by confocal microscopy, and *z*-projection was shown as the max intensity. Scale bar, 20 μm. The lower panels show statistical analysis of relative fluorescence intensity of control-cell body (C-C), control-migrasome (C-M), BAPTA-AM-cell body (B-C) and BAPTA-AM-migrasome (B-M). Data are means ± SEM. *n* > 100 cells from three independent experiments. Two-tailed unpaired *t*-test was used for statistical analyses. ***P < 0.001. **d** Diagram showing how migrasomes are involved in secretion in migrating cells.

If migrasomes serve as sites for cytokine secretion, the rate of migrasome formation might influence the amount of cytokines released by cells. To investigate this, we assessed the concentration of secreted cytokines in the culture medium of cell lines where migrasome formation was either enhanced or blocked. A previous study demonstrated that overexpressing Tspan4 significantly boosts migrasome formation.^30^ In this study, we observed that L929 cells stably overexpressing Tspan4 (T4 OE) exhibited significantly increased concentrations of M-CSF and CCL2 in their culture medium (Fig. 7a). We have previously shown that integrins are vital for migrasome formation.^31^ As anticipated, in Integrin β1 knockdown (Itg β1 KD) cells, migrasome formation was notably diminished. In these Itg β1 KD cells, concentrations of both M-CSF and CCL2 in the culture medium decreased, implying that migrasome formation is essential for the efficient secretion of M-CSF and CCL2 (Fig. 7a). Likewise, we discovered that overexpressing Myosin-5a increased, while knocking out Myosin-5a decreased, the concentration of M-CSF and CCL2 in the culture medium (Fig. 7a). This suggests that the transport of secretory vesicles plays a crucial role in the efficient secretion of cytokines.

### Migrasomes are the major sites of secretion in migrating cells

Cytokines within the migrasome are continuously secreted via SNARE-mediated fusion upon their transport into this structure. To accurately estimate the amount of cytokines released through the migrasome, we treated cells with BAPTA-AM for 10 h. This treatment blocks the fusion of secretory vesicles with both the migrasome membrane and the plasma membrane of the cell body. Subsequently, we stained L929 cells with M-CSF and CCL2 antibodies. We observed that blocking secretion significantly increased the quantity of cytokines in the migrasome, but not that in the cell body. In L929 cells after BAPTA-AM treatment, the levels of M-CSF and CCL2 increased to 9.5 and 10.6 times their original levels, respectively, within the migrasome. In contrast, the levels of M-CSF and CCL2 in the cell body only increased to 1.29 and 1.28 times the baseline values, respectively (Fig. 7b, c). Collectively, these results suggest that the secretion rate at the migrasome site is substantially higher than in the cell body, indicating that the migrasome is the primary secretion site for cytokines (Fig. 7d).

## Discussion

It is well established that migrasomes play their physiological roles through the targeted delivery of signaling molecules. One central question in the migrasome field is how signaling molecules are selectively transported into and released from migrasomes. Our study shows that secretory proteins, including signaling proteins, are delivered to migrasomes via secretory carriers from both constitutive and regulated secretion pathways. As cells move, many of these carriers are rerouted to the rear of the cell and actively moved into migrasomes, a process driven by the Myosin-5a motor protein. Upon reaching the migrasomes, these carriers fuse with the migrasome’s membrane through a fusion mediated by SNARE proteins. The fact that blocking the formation of migrasomes significantly reduces secretion levels indicates that migrasomes are a primary route for secretion in migrating cells. Our findings reveal a unique and localized method of secretion in migrating cells, akin to the targeted release of neurotransmitters in neuronal systems. In our initial paper detailing the discovery of the migrasome, we introduced the term “migracytosis” to describe the release of cellular content via the migrasome. Given the findings of our current study, we have refined the definition of “migracytosis” as a specialized secretion mode for migrating cells. In this mode, secretory proteins like cytokines are exocytosed through the migrasome.

In this study, we provide compelling evidence that migrasomes serve as the primary pathway for cytokine secretion in migrating cells. Our conclusions are bolstered by the following observations.

First, we demonstrate that migrasomes are significantly enriched with secretory cargoes. As illustrated, migrasomes exhibit markedly higher levels of secretory proteins compared to the cell body. This disparity suggests that secretory cargoes are preferentially concentrated within migrasomes. Second, the treatment of cells with BAPTA-AM, an inhibitor that blocks SNARE-mediated secretion both at the plasma membrane and within migrasomes, results in a more than tenfold increase in cytokine levels within the migrasomes. Conversely, there is a negligible elevation in cytokine levels within the cell body. This disparity indicates that the rate of secretion at the migrasome is significantly faster than at the cell body, highlighting migrasomes as the favored site of secretion in migrating cells. Finally, inhibiting the formation of migrasomes leads to a substantial reduction in the amount of cytokines secreted into the medium. This observation underscores the essential role of migrasomes in efficient secretion, suggesting that they are not only the preferred site but also a necessary component for effective cytokine release. Taken together, these findings strongly suggest that migrasomes constitute the main secretion route in migrating cells.

Migrasomes were initially categorized as organelles, distinct from extracellular vesicles, based on their biogenesis. Our study indicates that before detaching from the cell, migrasomes are secretion sites, performing essential cell-autonomous functions. After detachment, they act as extracellular vesicles, mediating long-distance cell communication. Thus, the migrasome’s lifecycle encompasses both intracellular and extracellular stages. First, while still connected to the cell, it serves as an organelle for localized secretion, similar to the synaptic terminals of neurons as both structures share a continuous plasma membrane and cytosol with the cell body. Once detached, it becomes an extracellular vesicle that facilitates cellular communication. This perspective underscores our belief that migrasomes are more akin to organelles than mere extracellular vesicles.

Numerous types of extracellular vesicles have been identified, and a crucial task in this field is distinguishing between different vesicle types. Our study provides markers for this purpose. We revealed that secretory carriers are sorted and transported into the migrasome in L929 cells and in DPSCs, leading to the enrichment of Myosin-5a, Rab8a, Rab11, VAMP2, VAMP3 and possibly other trafficking components. Since this vesicle trafficking process has not been observed in any other extracellular vesicle type, these proteins can serve as definitive markers to differentiate detached migrasomes from other vesicle types in these cell lines. Further work is needed to test whether the presence of secretory carriers is a common feature of migrasomes and whether these secretory components can be used as common markers for migrasomes derived from different cell types.

## Materials and methods

### Reagents and antibodies

Fibronectin (FN, #PHE0023), WGA (#W7024), Puromycin (#A1113803), Prolong Live Antifade Reagent (#P36975) and Lipofectamine 3000 (#L3000001) were purchased from Thermo Fisher Scientific. BAPTA-AM (#A1076), Diaminobenzidine (#D12384) and Proteinase K (#P8811) were purchased from Sigma-Aldrich. GLPG0187 (#HY-100506) was purchased from MCE. Vigofect (#T001) was purchased from Vigorous. G418 (#E859) was purchased from Amresco. Hygromycin B (#10843555001) was purchased from Roche.

Anti-Rab5 (#ab218624), anti-Rab8a (#ab188574), anti-Cellubrevin (#ab5789), anti-SNAP23 (#ab4114), anti-M-CSF (#ab233387) and anti-MCP1 (#ab7202) antibodies were from Abcam. Anti-Myosin-5a (#3402), anti-Rab8a (#6975), anti-Rab10 (#8127), anti-Rab11 (#5589), anti-VAMP2 (#13508), anti-Itg α5 (#4705) and anti-Itg β1 (#4706) antibodies were from Cell Signaling Technology. Anti-Cellubrevin (#ET7108-31) and anti-M-CSF (#ET1609-1) antibodies were from HuaAn Biotechnology. Anti-GAPDH (#60004-1-Ig) and anti-VAMP7 (#22268-1-AP) antibodies were from Proteintech Group. Anti-CCL2 (#AF-479-NA) antibody was from R&D SYSTEMS. Anti-syntaxin4 (#MA5-38156) antibody was from Thermo Fisher Scientific. Anti-CPQ (#HPA023235-100UL) antibody was from Sigma-Aldrich. Goat anti-Rabbit IgG (#111-035-003) and goat anti-mouse IgG (#115-035-003) were from Jackson.

### Cells

L929 cells were grown in Dulbecco’s Modified Eagle Medium (DMEM, #C11995500BT, Gibco) supplemented with 10% FBS (#04-001-1A, Biological Industries), 2 mM GlutaMAX (#35050-061, Gibco) and 100 U/mL penicillin–streptomycin (#GNM15140, GENOM). Cells were cultured at 37 °C in an incubator with 5% CO_2_.

DPSCs are a gift from Songling Wang’s Lab (Beijing Stomatological Hospital). DPSCs were grown in MEM-ALPHA (#C3060-0500, VivaCell) supplemented with 10% FBS, 2 mM Glutamax, 100 U/mL penicillin–streptomycin and 1% Antibiotic-Antimycotic (15240-062, Gibco). Cells were cultured at 37 °C in an incubator with 5% CO_2_.

### Cell transfection and virus infection

Cell transfection was conducted using Vigofect according to the manufacturer’s manual. The PiggyBac Transposon Vector System was used to generate stably expressing cell lines as previously described.^32^ Briefly, various proteins were cloned into pB-CAG (transposon vector) as the expressing plasmid backbone. The pB-CAG constructs combined with pBASE (transposase vector) were co-transfected into L929 cells at a ratio of 1:3 using the above Vigofect transfection protocol. After 24 h, the cells were treated with 600 µg/mL G418 or 200 µg/mL hygromycin B for selection (3–5 days). Single cells were sorted into 96-well plates by flow cytometry. These single-cell clones were cultured and expanded, followed by confocal analysis.

Gene knockdown was achieved with short hairpin RNA (shRNA) in the lentivirus-based vector pLKO.1-puro. Lentiviral production and infection were performed as previously described.^33^ Briefly, for lentiviral production, lentiviral vectors (pLKO.1, psPAX2 and pMD2.G) were co-transfected into 293T cells at a ratio of 4:3:1. After 48 h, the supernatant was centrifuged at 600× *g* for 5 min to remove cell debris. Viruses were harvested and used in the following experiments. For virus infection, the indicated cells seeded to 50%–60% confluence were co-cultured with virus containing 8 μg/mL polybrene for 24 h. The cells were placed in fresh medium containing 5 µg/mL puromycin for selection until drug-resistant colonies become visible. Sequences of the shRNAs were as follows: mouse *SNAP23*: 5′-GAACAACTAAATCGCATAGAA-3′, mouse *Stx4*: 5′-GAGTCCTGTCCCAGCAATTTG-3′, mouse *VAMP2*: 5′-CCTCAAGATGATGA-TCATCTT-3′, mouse *Itgβ1*: 5′-GCACGATGTGATGATTTAGAA-3′.

We used the CRISPR/Cas9-2hit KO system to generate the *Myosin-5a* knockout L929 cells. Two guide RNA (sgRNA) coding sequences were cloned into PX458M (5′-GTGCCGGTATGCGCCAGGCA-3′ and 5′-AGTTCGCTTCATCGATTCCA-3′). L929 cells were transfected with PX458M containing the *Myosin-5a*-targeting sequences. After single-cell sorting by flow cytometry, the single-cell clones were further analyzed by PCR and western blotting.

### Isolation of migrasomes from cultured cells

Crude migrasomes were collected by differential centrifugation as previously described.^34^ Briefly, cells and migrasomes in 15-cm dishes were gently harvested into 50-mL tubes after trypsin digestion. All subsequent manipulations were conducted at 4 °C. After double centrifugation at 600× *g* for 10 min at 4 °C, the supernatant was further centrifuged at 2000× *g* for 20 min at 4 °C to remove the cell bodies and large debris. Crude migrasomes were then acquired as the pellet by centrifugation at 18,000× *g* for 30 min at 4 °C.

High-purity migrasome isolation was performed by iodixanol–sucrose (#LYSISO1, Sigma-Aldrich) density gradient centrifugation following a published protocol with minor modifications.^35^ Briefly, the crude migrasome pellet was resuspended in 800 µL buffer (400 µL extraction buffer mixed with 400 µL 10% Optiprep) and then fractionated at 150,000× *g* for 4 h at 4 °C in a multistep Optiprep dilution gradient. The step gradient was 50% (500 µL), 40% (500 µL), 35% (500 µL), 30% (500 µL), 25% (500 µL), 20% (500 µL), 15% (500 µL), 10% (500 µL), 5% (500 µL) and crude migrasomes (5%, 800 µL). After centrifugation, samples were collected from top to bottom gently (500 µL per fraction). Fractions 4, 5 and 6 were each mixed with 500 μL PBS and then centrifuged at 18,000× *g* for 30 min at 4 °C. The pellets were washed with PBS and centrifuged again at 18,000× *g* for 30 min to pellet the migrasomes. The samples were immediately available for downstream applications such as western blot and TEM analyses.

### Imaging and image analysis

10 µg/mL FN was used to precoat confocal dishes at 37 °C for at least 1 h. For confocal snapshot images, cells were cultured in FN-precoated confocal dishes for 10–12 h, and imaged by a NIKON A1RSiHD25 laser scanning confocal microscope at 1024 × 1024 pixels. *Z*-stack imaging of cells and migrasomes was performed with a NIKON A1 microscope. SIM images were acquired using a Nikon N-SIM Super Resolution Microscope.

For long-term time-lapse images, cells were grown in FN-precoated confocal dishes for 4–6 h before imaging. Cells were then maintained in the living cell system (37 °C, 5% CO_2_), and monitored by a NIKON A1 microscope. Ultra-fast super-resolution time-lapse images were collected using a GI-SIM. NIS-Elements analysis 5.4 software was used to deconvolute images acquired by the NIKON A1 microscope. *Z*-projection and 3D reconstruction were performed with NIS-Elements 5.4. Images were processed using ImageJ and Imaris software 8.1.4, and statistical analyses were conducted by GraphPad Prism 8.

### TEM

The preparation of TEM samples was conducted following the protocol that we set up previously.^32^ Briefly, cells were grown in 35-mm dishes precoated with FN (10 µg/mL) for 10–12 h. Cells were pre-fixed with a 1:1 mixture of growth medium and 2.5% glutaraldehyde for 5 min, and were further fixed in 2.5% glutaraldehyde in PB buffer for 2 h at room temperature. After three gentle washes with PBS, cells were dehydrated through a graded ethanol series (50%, 70%, 90%, 95% and 100%) for 8 min per step. The samples were subsequently infiltrated and embedded in SPON12 resin, polymerized at 60 °C for 48 h. Ultrathin 70-nm sections were cut with a diamond knife, collected on Formvar-coated copper grids (100 mesh). These sections were then double-stained with uranyl acetate and lead citrate. After air-drying, samples were examined using an H-7650B TEM at 80 kV.

For APEX2-based intracellular-specific protein imaging by TEM, the procedure was based on the previous protocol with minor modifications.^36^ Briefly, cells were fixed with 2% glutaraldehyde (in 100 mM sodium cacodylate buffer with 2 mM CaCl_2_, pH 7.4) at room temperature, then moved to ice for 1 h. All subsequent manipulations were conducted at 4 °C until resin infiltration. After three gentle washes with chilled buffer, cells were treated with 20 mM glycine for 5 min to quench unreacted glutaraldehyde. A freshly diluted DAB solution (0.5 mg/mL DAB in HCl combined with 0.03% H_2_O_2_) was added to cells for 5 min. The local deposition of DAB catalyzed by APEX2 could be monitored by light microscopy, and the reaction was stopped by washing three times with chilled buffer. Post-fixation staining with 2% osmium tetroxide (#1250423, SPI) was conducted for 30 min in chilled buffer, followed by washing and soaking in 2% uranyl acetate (#22400, Electron Microscopy Sciences) overnight. Dehydration was performed in cold ethanol (20%, 50%, 70%, 90%, 100%, 100%) for 2 min per step, followed by infiltration with Durcupan ACM resin (Electron Microscopy Sciences) mixed with anhydrous ethanol (1:1) for 30 min, then 100% resin twice for 1 h each. Samples were embedded in fresh resin and polymerized under vacuum at 60 °C for 48 h. DAB-stained areas of embedded cultured cells were identified using transmitted light. After sawed out with a jeweler’s saw, the areas of interest were mounted on dummy acrylic blocks with cyanoacrylic adhesive (Krazy Glue, Elmer’s Products). Ultrathin 70-nm sections were cut as described above, and samples were examined using an H-7650B TEM at 80 kV.

### Total membrane protein isolation

Total membrane proteins from plasma membranes and from migrasomes were isolated using the Pierce Cell Surface Protein Isolation Kit (#89881, Thermo Fisher Scientific) according to the manufacturer’s instructions. Briefly, cells were cultured in FN-precoated confocal dishes for 10 h or 12 h. After being washed with PBS, cells were then treated with Sulfo-NHS-SS-Biotin to biotinylated cell membrane proteins. Biotin-labeled membrane proteins were subsequently isolated from cell bodies or migrasomes using NeutrAvidin Agarose, respectively. Membrane proteins from cell bodies and migrasomes were normalized to equal total protein loading for western blot analysis.

### Secretion analysis

Cargo secretion analysis was conducted following a published protocol with minor modifications.^37^ Briefly, equal numbers of the indicated cells were seeded into FN-precoated dishes for 10 or 16 h, and the medium was gently collected into 50 mL tubes. After double centrifugation at 600× *g* for 10 min at 4 °C, the supernatant was further centrifuged at 2000× *g* for 20 min at 4 °C to remove the cell bodies and large debris. Soluble proteins in the medium were concentrated by a 10 KD Amicon filter (Millipore), and the cell lysates were collected, respectively. These concentrated medium was normalized with the numbers of cells, and then subjected to western blot analysis.

### Western blot analysis

The details of western blot analysis were described before.^32^ Briefly, the indicated cells or migrasomes were lysed by 2.5% SDS lysis buffer and boiled for 10–20 min at 95 °C. The protein concentration of each sample was determined using the BCA kit. Proteins were separated on SDS–PAGE gels of an appropriate percentage according to the molecular weight of the target proteins, followed by electrophoretic transfer onto PVDF membranes. After blocking with 5% non-fat milk in TBST buffer, membranes were incubated with primary antibody overnight at 4 °C. Membranes were then incubated with secondary antibody (HRP) for 1 h at room temperature, and signals were detected with a WESTAR ηC 2.0 kit (CYANAGEN).

The following primary antibodies were used for western blot analysis at the indicated dilution: anti-Myosin-5a (1:1000), anti-SNAP23 (1:2000), anti-M-CSF (1:1000), anti-CCL2 (1:1000), anti-PIGK (1:1000), anti-CPQ (1:1000), anti-Itg α5 (1:1000), anti-Itg β1 (1:1000), anti-Syntaxin4 (1:1000), anti-VAMP2 (1:1000) and anti-GAPDH (1:5000).

### Statistical analysis

Statistical analyses were conducted using the unpaired two-tailed *t*-test in Graphpad Prism 5 (or 8) software (Graphpad Software). Data are the means ± SEM. Significance is indicated by asterisks: *P < 0.05, **P < 0.01, ***P < 0.001, ****P < 0.0001, NS, not significant. Statistical parameters and significance are reported in the figures and the figure legends.

### Supplementary Information

Supplementary information, Fig. S1

Supplementary information, Fig. S2

Supplementary information, Fig. S3

Supplementary information, Fig. S4

Supplementary information, Video S1

Supplementary information, Video S2

Supplementary information, Video S3

Supplementary information, Video S4

Supplementary information, Video S5

Supplementary information, Video S6

Supplementary information, Video S7

Supplementary information, Video legend
