# Supplementary material for: Localized, highly efficient secretion of signaling proteins by migrasomes
Source: Cell Res. 2024 Jun 25;34(8):572–85. doi: 10.1038/s41422-024-00992-7 (PMC11291916; doi:10.1038/s41422-024-00992-7)
Supplement: Supplementary file 1 — Supplementary information, Fig. S1 [file 41422_2024_992_MOESM1_ESM.pdf]

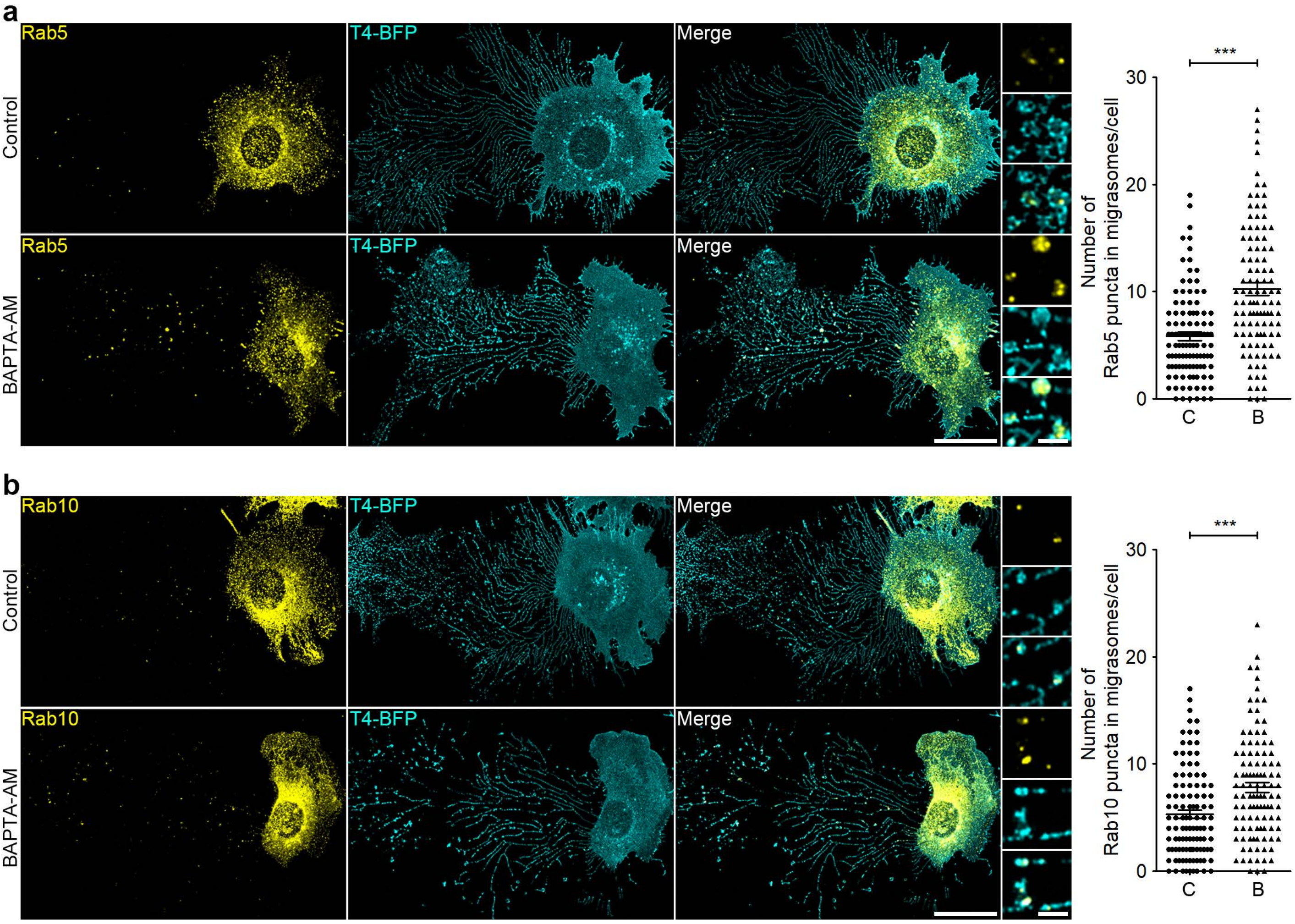
a
Rab5
T4-BFP
Merge
Control
BAPTA-AM
***
Number of
Rab5 puncta in migrasomes/cell
0
10
20
30
C
B
b
Rab10
T4-BFP
Merge
Control
BAPTA-AM
***
Number of
Rab10 puncta in migrasomes/cell
0
10
20
30
C
B

Figure S1

Fig. S1 Rab5- and Rab10-labeled vesicles fuse with the migrasome membranes in a calcium-dependent manner.

a, b Immunostaining of endogenous Rab5 (**a**) or Rab10 (**b**) in L929-T4-BFP cells treated with 10 μM BAPTA-AM for 10 hr. Scale bar, 20 μm. Middle panels, enlarged ROI. Scale bar, 2 μm. Right panel, statistical analysis of the number of Rab5 (**a**) or Rab10 (**b**) puncta in migrasomes per cell. Error bars, mean ± SEM. $n > 100$ cells from three independent experiments. Two-tailed unpaired t-test was used for statistical analyses. ***$p < 0.001$.
