# Supplementary material for: Localized, highly efficient secretion of signaling proteins by migrasomes
Source: Cell Res. 2024 Jun 25;34(8):572–85. doi: 10.1038/s41422-024-00992-7 (PMC11291916; doi:10.1038/s41422-024-00992-7)
Supplement: Supplementary file 2 — Supplementary information, Fig. S2 [file 41422_2024_992_MOESM2_ESM.pdf]

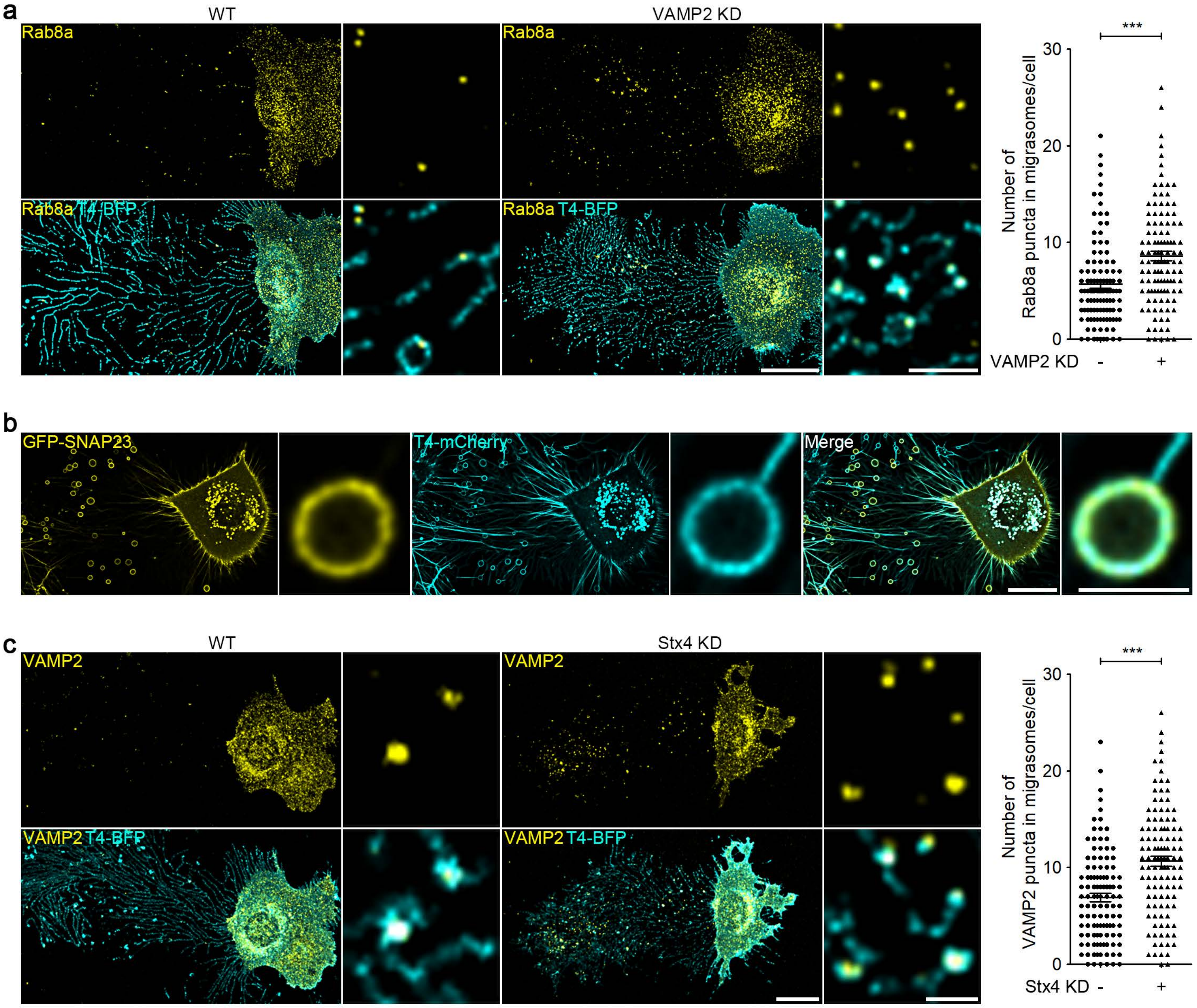
a
WT
VAMP2 KD
Rab8a
Rab8a T4-BFP
***
Number of
Rab8a puncta in migrasomes/cell
30
20
10
0
VAMP2 KD - +
b
GFP-SNAP23
T4-mCherry
Merge
c
WT
Stx4 KD
VAMP2
VAMP2 T4-BFP
***
Number of
VAMP2 puncta in migrasomes/cell
30
20
10
0
Stx4 KD - +

Figure S2

Fig. S2 SNAREs are required for the fusion of intraluminal vesicle with the migrasome membrane.

a L929-T4-BFP cells were infected with nonspecific (WT) or VAMP2-shRNA (VAMP2 KD) lentiviral constructs. Cells were then immunostained with Rab8a antibody and subjected to confocal analysis. Scale bar, 20 µm. Right panels, enlarged ROI. Scale bar, 2 µm. Statistical analysis of the number of Rab8a puncta in migrasomes per cell is shown as the mean ± SEM. $n > 100$ cells from three independent experiments were analyzed using the two-tailed unpaired t-test. ***$p < 0.001$.

b Confocal images of L929 cells stably expressing GFP-SNAP23 and T4-mCherry. Scale bar, 20 µm. The right panels show enlarged migrasome. Scale bar, 2 µm.

c Immunostaining of endogenous VAMP2 in WT or syntaxin4 (Stx4) KD L929-T4-BFP cells. Scale bar, 20 µm. Right panels, enlarged ROI. Scale bar, 2 µm. Statistical analysis of the number of VAMP2 puncta in migrasomes per cell is shown as the mean ± SEM. $n > 100$ cells from three independent experiments were analyzed using the two-tailed unpaired t-test. ***$p < 0.001$.
