# Supplementary material for: Localized, highly efficient secretion of signaling proteins by migrasomes
Source: Cell Res. 2024 Jun 25;34(8):572–85. doi: 10.1038/s41422-024-00992-7 (PMC11291916; doi:10.1038/s41422-024-00992-7)
Supplement: Supplementary file 3 — Supplementary information, Fig. S3 [file 41422_2024_992_MOESM3_ESM.pdf]

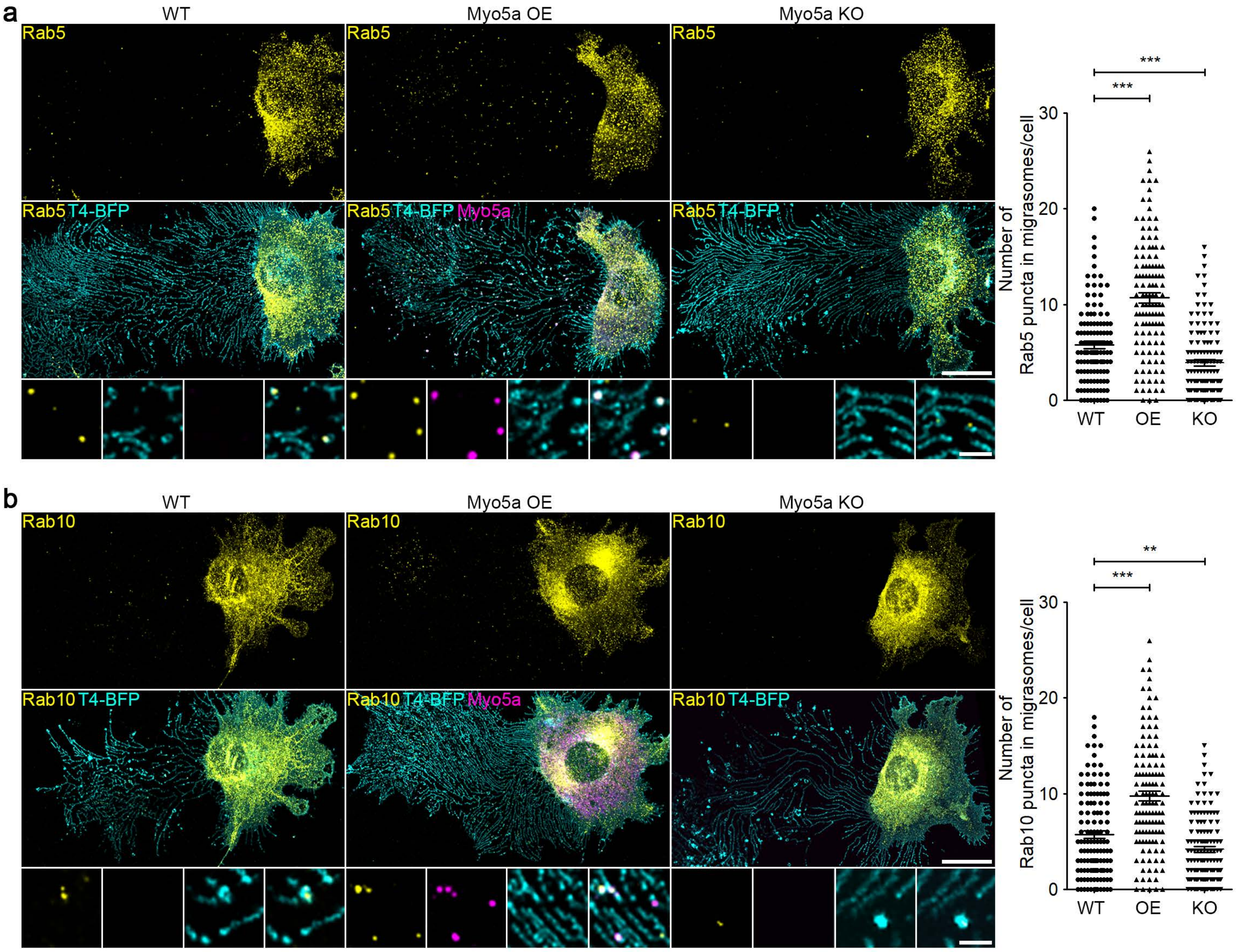
a
WT
Myo5a OE
Myo5a KO
Rab5
Rab5 T4-BFP
Rab5 T4-BFP Myo5a
Number of Rab5 puncta in migrasomes/cell
30
20
10
0
WT
OE
KO
***
***
b
Rab10
Rab10 T4-BFP
Rab10 T4-BFP Myo5a
Number of Rab10 puncta in migrasomes/cell
**
***

Figure S3

Fig. S3 Rab5- and Rab10-labeled vesicles are transported into migrasomes by Myosin5a.

a, b Immunostaining of endogenous Rab5 (**a**) or Rab10 (**b**) in WT, Myo5a OE and Myo5a KO L929-T4-BFP cells. Scale bar, 20 μm. Lower panels, enlarged ROI. Scale bar, 2 μm. Right panel, statistical analysis of the number of Rab5 (**a**) or Rab10 (**b**) puncta in migrasomes per cell. Error bars, mean ± SEM. $n > 100$ cells from three independent experiments. Two-tailed unpaired t-test was used for statistical analyses. $^{**}p < 0.01$, $^{***}p < 0.001$.
