# Supplementary material for: Localized, highly efficient secretion of signaling proteins by migrasomes
Source: Cell Res. 2024 Jun 25;34(8):572–85. doi: 10.1038/s41422-024-00992-7 (PMC11291916; doi:10.1038/s41422-024-00992-7)
Supplement: Supplementary file 4 — Supplementary information, Fig. S4 [file 41422_2024_992_MOESM4_ESM.pdf]

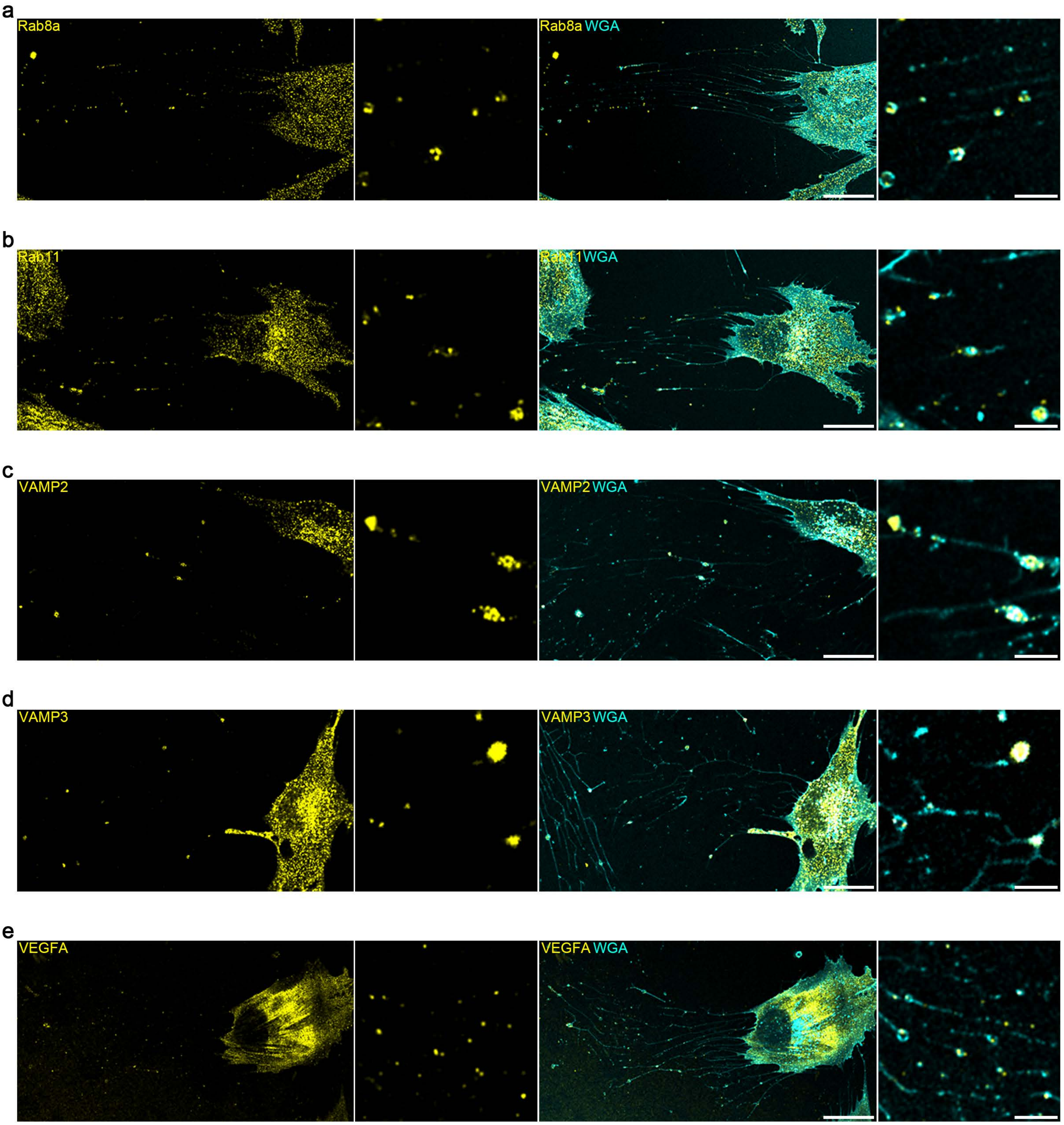
a
Rab8a
Rab8a WGA
b
Rab11
Rab11 WGA
c
VAMP2
VAMP2 WGA
d
VAMP3
VAMP3 WGA
e
VEGFA
VEGFA WGA

Figure S4

Fig. S4 DPSCs and L929 cells utilize similar pathway for migrasome-mediated secretion.

a-e Immunostaining of endogenous Rab8a (**a**), Rab11 (**b**), VAMP2 (**C**), VAMP3 (**d**) and VEGFA (**e**) in DPSCs. Scale bar, 20 μm. The right panels show enlarged migrasome. Scale bar, 2 μm.
