# Supplementary material for: Localized, highly efficient secretion of signaling proteins by migrasomes
Source: Cell Res. 2024 Jun 25;34(8):572–85. doi: 10.1038/s41422-024-00992-7 (PMC11291916; doi:10.1038/s41422-024-00992-7)
Supplement: Supplementary file 12 — Supplementary information, Video legend [file 41422_2024_992_MOESM12_ESM.pdf]

Supplementary video titles and legends

Video S1 Continuous calcium signal within the migrasome during its formation.

L929-T4-BFP cells were stained with Fluo-8 and then subjected to time-lapse imaging. Time interval, 180 s. Scale bar, 10 μm.

Video S2 VAMP2 vesicles fuse with the migrasome membrane during migrasomes grow.

L929-GFP-VAMP2 cells were subjected to time-lapse imaging. Time-lapse images were acquired at intervals of 30 s. Scale bar, 2 μm.

Video S3 Myosin5a transports to the site of migrasome formation.

Time-lapse imaging of L929 cells stably expressing GFP-Myo5a and T4-mCherry. Time interval, 90 s. Scale bar, 20 μm.

Video S4 Two modes of the movement of Myosin5a transported into migrasomes.

Grazing Incidence-Structured Illumination Microscopy (GI-SIM) imaging of L929- GFP-Myo5a cells. Time-lapse images were acquired at intervals of 30 s. Scale bar, 5 μm.

Video S5 Myosin5a displays polarized distribution during cell migration.

L929 cells stably expressing mCherry-Myo5a and T4-BFP, treated with or without 10 μM GLPG0187, were subjected to time-lapse imaging. Time interval, 10 min. Scale bar, 20 μm.

Video S6 Cell migration causes the polarization of Rab8a vesicles to the rear end of the cell. L929 cells stably expressing GFP-Rab8a and T4-BFP, treated with or without 10 μM GLPG0187, were subjected to time-lapse imaging. Time interval, 10 min. Scale bar, 20 μm.

Video S7 Cell migration causes the polarization of Rab11a vesicles to the rear end of the cell. L929 cells stably expressing GFP-Rab11a, treated with or without 10 μM GLPG0187, were stained with WGA and then subjected to time-lapse imaging. Time interval, 10 min. Scale bar, 20 μm.
